# Trends and factors associated with cyber fraud among older adults: a decade of administrative data in Brazil

**DOI:** 10.3389/fpubh.2026.1884877

**Published:** 2026-07-15

**Authors:** Thaynara Ferreira Lopes, Paulo César de Almeida, Aridiane Alves Ribeiro, Meiry Fernanda Pinto Okuno

**Affiliations:** 1 Department of Nursing, Federal University of São Paulo, São Paulo, Brazil; 2 Department of Nursing, State University of Ceará, Fortaleza, Brazil

**Keywords:** aged, Brazil, epidemiology, financial exploitation, fraud, internet

## Abstract

**Background:**

Cyber fraud targeting older adults has become an increasingly important public health and social concern, particularly in low- and middle-income countries. Despite growing digital inclusion, older populations remain disproportionately exposed to financial exploitation in online environments. This study aimed to examine temporal trends and identify factors associated with cyber fraud among older adults using administrative data from Brazil.

**Methods:**

A cross-sectional, retrospective study was conducted using administrative police records of reported cyber fraud cases involving individuals aged ≥60 years in the state of Ceará, Brazil, between 2015 and 2025. Descriptive analyses were performed to characterize temporal trends and victim profiles. Bivariate analyses and multivariable logistic regression models were applied to identify factors associated with the occurrence and distribution of cases. Variables included sex, age, geographic region, and contextual characteristics of reported incidents.

**Results:**

A total of 12,413 cases were identified over the study period, with a marked increase observed from 2020 onwards. The highest proportions of cases occurred in 2023 (24.4%) and 2024 (30.8%), followed by a decline in 2025, likely reflecting incomplete data. Trend analyses were therefore restricted to the complete period from 2019 to 2024. Most cases were concentrated in the capital, Fortaleza (65.7%), followed by the metropolitan region (9.8%) and inland areas (24.5%). Women were more frequently affected (55.0%), and the mean age of victims was 67.1 years (SD = 5.9). Multivariable analysis indicated that female sex, residence in urban areas, and more recent years were associated with increased reporting of cyber fraud.

**Conclusions:**

Cyber fraud against older adults has increased substantially over the past decade, with distinct demographic and regional patterns. These findings underscore the need for targeted public health strategies, digital literacy interventions, and intersectoral policies to protect older populations from financial exploitation in digital environments. Strengthening surveillance systems and improving reporting mechanisms are essential to support prevention and response efforts.

## Background

Population aging, combined with the rapid expansion of digital technologies, has substantially reshaped social, economic, and communication dynamics worldwide. In this context, the growing participation of older adults in digital environments has been accompanied by emerging challenges related to information security, particularly increased exposure to cyber fraud and other forms of cybercrime. Evidence suggests that while digital connectivity promotes social inclusion and access to services, it simultaneously increases vulnerability to online scams, especially those based on social engineering and behavioral manipulation (1).

This phenomenon became more pronounced during the COVID-19 pandemic, which accelerated the digitalization of financial, healthcare, and communication services. As a result, older adults were increasingly exposed to digital environments, often without the parallel development of adequate digital safety skills. International studies indicate that this shift contributed to a rise in cyber fraud targeting older populations, reinforcing its characterization as a growing public health and societal concern (2).

Although older adults are often perceived as inherently more vulnerable to digital fraud, recent research highlights that this vulnerability is multifactorial and cannot be explained by age alone. Factors such as digital literacy, cognitive status, social isolation, and patterns of online interaction play a critical role in shaping susceptibility to fraud (3). Moreover, the increasing sophistication of cyber scams—including those involving artificial intelligence and deepfake technologies—has further reduced the effectiveness of traditional risk detection strategies, expanding vulnerability across different population groups (4).

At the global level, research on cyber fraud affecting older adults has advanced, particularly in high-income countries. However, important gaps persist in low- and middle-income settings, where rapid digital expansion often occurs without the parallel development of structured digital protection policies. In such contexts, the intersection of population aging, socioeconomic inequalities, and accelerated digitalization may amplify exposure to cybercrime, making this issue particularly relevant from a public health perspective (5).

In Brazil, despite the growing number of reported cybercrime cases, studies using administrative public security data to specifically examine cyber fraud among older adults remain limited. Most available research relies on self-reported data or restricted samples, limiting the understanding of the magnitude and characteristics of this phenomenon at the population level. Consequently, there is a critical need for studies that systematically analyze official records over time (6).

Therefore, this study aims to analyze temporal trends and identify factors associated with cyber fraud against older adults in the state of Ceará, Brazil, from 2015 to 2025, using administrative public security data. By leveraging a comprehensive institutional database, this study provides robust empirical evidence on the dynamics of cyber fraud in a middle-income country context, contributing to the international literature on digital security and population aging.

## Methods

### Study design and setting

A cross-sectional, retrospective study was conducted using a quantitative approach based on documentary analysis of police reports related to cyber fraud cases involving older adults in the state of Ceará, Brazil, from 2015 to 2025.

### Data source and study population

This study is part of a broader research project entitled “*Empowering to protect: development of a social technology prototype for the digital security of older adults*.” Data were obtained from the institutional database of the Ceará State Secretariat of Public Security and Social Defense, through the Superintendence for Research and Public Security Strategy, specifically the Directorate of Statistics and Geoprocessing.

This secondary database compiles official police records systematically organized for monitoring and statistical purposes in public security and is accessible for scientific use upon formal request.

Police reports involving individuals aged ≥60 years residing in the state of Ceará who were victims of cyber fraud during the study period were included. Records with incomplete or insufficient information for the variables of interest were excluded.

### Data collection and variables

Secondary data were collected by the principal investigator between February and April 2026. A formal request was submitted to obtain variables including year of occurrence, municipality of residence, sex, education level, marital status, race/ethnicity, age, characteristics of the fraud, offender profile, and means used to commit the crime (e.g., phone calls, emails, and messages).

Due to the sensitive nature of public security data, access was restricted to aggregated and anonymized information, ensuring that no individual-level identification was possible.

### Statistical analysis

Descriptive analyses were performed using absolute and relative frequencies for categorical variables, and measures of central tendency and dispersion (mean and standard deviation) for continuous variables.

Associations between categorical variables were assessed using Pearson's chi-square test. Multivariable logistic regression models were applied to identify factors associated with cyber fraud occurrence, considering sociodemographic and territorial variables as independent predictors.

Temporal trends were evaluated using Pearson's correlation analysis, followed by simple linear regression. To better represent the period of sustained growth in cyber fraud notifications, trend analyses were restricted to the complete period from 2019 to 2024. Data from 2025 were excluded because the year was incomplete at the time of extraction, and records before 2019 were sporadic.

All analyses were performed using the Statistical Package for the Social Sciences (SPSS), adopting a significance level of 5% (*p* < 0.05) and a 95% confidence interval.

### Ethical considerations

This study used secondary, aggregated, and anonymized data with no possibility of individual identification. Therefore, ethical approval was waived in accordance with Brazilian regulations, including Resolution No. 466/2012 of the National Health Council and related guidelines governing the use of institutional data for research purposes.

## Results

A total of 12,413 police reports of virtual fraud involving older adults were analyzed in the state of Ceará, Brazil, from 2015 to 2025. A marked increase in the number of cases was observed over time, particularly from 2020 onwards. The highest proportions were recorded in 2023 (24.4%) and 2024 (30.8%). In 2025, a reduction in the number of cases was identified, possibly due to the incompleteness of recent data.

Regarding territorial distribution, most cases were concentrated in the capital, Fortaleza, which accounted for 65.7% of all occurrences, followed by the metropolitan region (9.8%) and inland municipalities (24.5%). When stratified by population size, 15.2% of cases occurred in municipalities with fewer than 75,000 inhabitants, while 9.2% were recorded in municipalities with larger populations.

In terms of the sociodemographic profile, a higher proportion of victims were female (55%) compared to male (45%). The mean age was 67.08 years (standard deviation = 5.92), with a predominance of individuals aged 60–64 years (40.7%), followed by those aged 65–69 years (30.1%). Additional sociodemographic characteristics are presented in Table 1.

**Table 1:** Sociodemographic characteristics of older adults who were victims of virtual fraud in Ceará, Brazil, 2015–2025 (*n* = 12,413).

Variable	*n*	%
Sex		
Female	6,789	55.0
Male	5,545	45.0
Age group (years)		
60–64	5,051	40.7
65–69	3,742	30.1
70–74	2,190	17.6
≥75	1,430	11.5
Education level		
Illiterate	304	2.7
Literate	1,645	14.5
Primary education	1,667	14.7
Secondary education	3,184	28.2
Higher education	4,508	39.9
Marital status		
Married	6,326	53.9
Single	2,145	18.3
Separated/divorced	1,752	14.9
Widowed	1,510	12.9

Source: Prepared by the authors.

Regarding educational level, a higher frequency of victims with higher education was observed (39.9%), followed by those with secondary education (28.2%).

In terms of marital status, most victims were married (53.9%), followed by single individuals (18.3%), separated/divorced (14.9%), and widowed (12.9%).

The analysis of the association between sociodemographic variables and victims' sex is presented in Table 2. No statistically significant association was found between age group and sex (*p* = 0.201). In contrast, statistically significant associations were identified between marital status and sex (*p* < 0.0001), as well as between place of residence and sex (*p* = 0.002). Notably, among men, 69.4% were married, whereas this proportion was 41.1% among women.

**Table 2:** Association between sociodemographic characteristics and sex of victims of virtual fraud in Ceará, Brazil, 2015–2025.

Variable	Female *n* (%)	Male *n* (%)	Total *n* (%)	*p*-value
Age group (years)				0.201
60–64	2,817 (41.5)	2,196 (39.6)	5,013 (40.6)	
65–69	2,023 (29.8)	1,699 (30.6)	3,722 (30.2)	
70–74	1,183 (17.4)	994 (17.9)	2,177 (17.7)	
≥75	766 (11.3)	656 (11.8)	1,422 (11.5)	
Marital status				< 0.001
Married	2,644 (41.1)	3,676 (69.4)	6,320 (53.9)	
Separated/divorced	1,079 (16.8)	673 (12.7)	1,752 (14.9)	
Single	1,480 (23.0)	661 (12.5)	2,141 (18.3)	
Widowed	1,225 (19.1)	285 (5.4)	1,510 (12.9)	
Place of residence				0.002
Fortaleza	4,545 (66.9)	3,549 (64.0)	8,094 (65.6)	
Metropolitan region	621 (9.1)	592 (10.7)	1,213 (9.8)	
Inland (>75,000 inhabitants)	626 (9.2)	517 (9.3)	1,143 (9.3)	
Inland ( ≤ 75,000 inhabitants)	997 (14.7)	887 (16.0)	1,884 (15.3)	

Source: Prepared by the authors.

**Figure 1:**
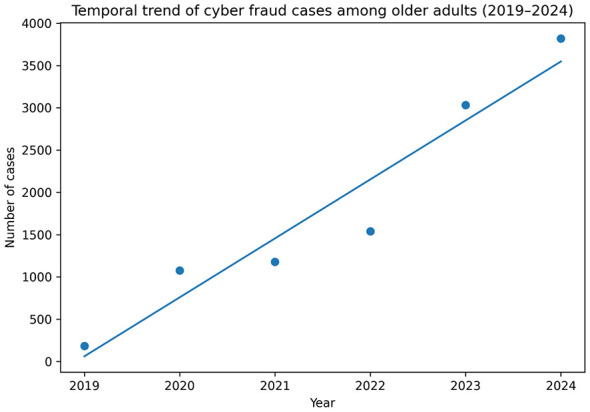
Temporal trend of cyber fraud cases involving older adults in Ceará, Brazil, 2019–2024. Source: Prepared by the authors.

The temporal trend of virtual fraud cases involving older adults is presented in Figure 1. A progressive increase in the number of reported cases was observed over the study period, with a more pronounced rise from 2020 onwards.

Only 22 cases were identified between 2015 and 2018, indicating sporadic reporting during the early years of the series. Therefore, temporal trend analyses were restricted to the period from 2019 to 2024, when a sustained increase in cyber fraud notifications was observed. During this period, the number of reported cases increased substantially, from 184 cases in 2019 to 3,818 cases in 2024.

Pearson's correlation analysis demonstrated a strong positive association between the number of reported cases and calendar year (*r* = 0.963; *p* = 0.002). Simple linear regression indicated an average annual increase of approximately 697 cases (β = 697.1 cases/year; *R*^2^ = 0.926; *p* = 0.002).

These findings demonstrate a continuous and accelerated expansion of virtual fraud cases involving older adults in the state of Ceará, particularly in the most recent years of the analyzed time series.

## Discussion

The findings of this study demonstrate a substantial increase in cyber fraud cases involving older adults over the analyzed period, with a more pronounced rise from 2020 onwards. This pattern is consistent with international evidence observed during the COVID-19 pandemic, a period marked by accelerated digitalization and increased reliance on online services among populations previously less engaged in digital environments. This scenario has been described as a “window of opportunity” for fraud, in which increased digital exposure coincided with limited preparedness among older users (7).

The strong temporal trend identified in this study reinforces evidence that cyber fraud targeting older adults is not only increasing in frequency but also becoming more sophisticated and targeted. Recent studies indicate that perpetrators increasingly rely on psychological manipulation strategies—such as exploiting trust, urgency, and authority—rather than solely technical vulnerabilities (8).

In this context, the present study contributes to the literature by providing empirical evidence based on real-world administrative data in a middle-income country. This represents an important advancement, as much of the existing literature is based on self-reported or experimental data, which may be subject to reporting and perception biases (6).

The concentration of cases in urban areas, particularly in the capital, is consistent with studies demonstrating higher incidence of digital fraud in settings with greater connectivity and broader use of digital financial services (9). However, the presence of a considerable proportion of cases in inland municipalities suggests a process of diffusion of digital vulnerability, indicating that the expansion of connectivity has not been accompanied by equivalent investments in digital literacy and protection strategies. This finding is particularly relevant for low- and middle-income countries, where technological expansion often occurs unevenly (10).

Regarding the profile of victims, the higher proportion of women may reflect broader demographic patterns, including the feminization of aging. However, recent evidence suggests that vulnerability to cyber fraud is not inherently determined by sex, but rather influenced by contextual factors such as social support networks, patterns of interaction, and levels of digital engagement (11).

One of the most relevant findings of this study is the high proportion of victims with higher levels of education. This result challenges traditional assumptions that associate vulnerability primarily with low educational attainment and aligns with emerging evidence indicating that susceptibility to fraud is more closely related to behavioral and cognitive factors, such as overconfidence, decision-making heuristics, and risk perception (12).

The association between marital status and sex, although expected from a demographic perspective, may also be interpreted in light of psychosocial factors. Conditions such as loneliness and social isolation have been consistently associated with increased vulnerability to fraud among older adults, as they may enhance susceptibility to emotionally driven or trust-based scams (13).

The strong increase observed between 2019 and 2024, corresponding to approximately 697 additional reported cases per year, supports the interpretation of cyber fraud against older adults as an expanding public health and social problem (14).

The very small number of cases observed between 2015 and 2018 should be interpreted with caution. These findings may reflect lower digital engagement among older adults during the early years of the series, reduced awareness of cyber fraud, and possible underreporting within police information systems. From 2019 onwards, the sharp increase in reported cases coincided with the growing adoption of digital technologies, online banking services, and internet use among older adults. This trend became even more pronounced during the COVID-19 pandemic, when social distancing measures accelerated the use of digital platforms for communication, financial transactions, and access to services. For this reason, data from 2025 were excluded from temporal trend modeling.

From a methodological perspective, this study is strengthened by the use of administrative public security data, which allows for a more accurate representation of officially recorded events. Compared to self-reported studies, such data reduce recall and perception biases, although they may still underestimate the true magnitude of the phenomenon due to underreporting (15).

Some limitations should be acknowledged. The use of aggregated data precludes individual-level analyses and limits the ability to control for potential confounding factors. In addition, the lack of detailed information on financial losses, types of fraud, and perpetrator characteristics restricts a more comprehensive understanding of the phenomenon. Nevertheless, the large sample size and extended time series enhance the robustness of the findings.

From a public health perspective, the results highlight the need for intersectoral strategies integrating public security, health, and social services, with a focus on developing digital security competencies among older adults. Evidence suggests that targeted educational interventions, combined with accessible reporting mechanisms and institutional support, may reduce vulnerability to fraud (5).

Finally, these findings have broader international relevance, particularly for countries undergoing rapid digital transformation alongside population aging. Understanding the dynamics of cyber fraud in diverse contexts is essential for informing global prevention strategies tailored to different sociocultural and economic settings.

## Conclusion

This study contributes to advancing current knowledge by examining the dynamics of cyber fraud against older adults using administrative public security data in a context of rapidly expanding digital connectivity. The findings highlight that vulnerability in digital environments extends beyond traditional factors, such as low educational attainment or limited access, encompassing behavioral, social, and structural dimensions.

In this regard, the results underscore the need to reorient prevention strategies, with a focus on developing specific competencies related to digital security. These strategies should go beyond formal literacy and incorporate aspects such as risk perception, decision-making processes, and patterns of interaction in online environments.

From a public policy perspective, the findings emphasize the importance of intersectoral approaches that integrate public security, health, and social services, as well as the strengthening of institutional mechanisms for victim protection and support. Such strategies are essential to address the increasing sophistication of digital crimes and to reduce the exposure of older adults to financial exploitation.

Finally, future research should prioritize individual-level and longitudinal analyses, as well as the inclusion of variables related to types of fraud, financial impact, and perpetrator characteristics, in order to deepen the understanding of this phenomenon and support the development of more targeted and effective interventions.

## Data Availability

The datasets analyzed in this study are not publicly available because they contain administrative data provided by the Ceará State Secretaof Public Security and Social Defense (SSPDS/CE) under institutional authorization. Requests to access these datasets should be directed to the corresponding public authority, subject to institutional approval and applicable legal.
